# Community pharmacy-based alcohol brief intervention in the UK: significant alcohol consumption reduction in risky drinkers

**DOI:** 10.1186/1940-0640-7-S1-A55

**Published:** 2012-10-09

**Authors:** Natasha Khan, Ranjita Dhital, Cate Whittlesea, Ian Norman, Peter Milligan

**Affiliations:** 1 Institute of Pharmaceutical Science, King’s College London, London, UK; 2 Division of Health and Social Care Research, King's College London, London, UK; 3 School of Biomedical and Health Sciences, King's College London, London, UK

## 

Previous studies have shown that community-pharmacy–based screening and brief intervention (SBI) for risky alcohol use is feasible. However, few studies have reported significant reductions in alcohol use following pharmacy-delivered BI. In this study, trained pharmacists (N = 29) at 28 community pharmacies in London, UK, offered BI from February-July 2010. Customers seeking alcohol-related medication and/or advice were targeted. Participating pharmacists used Alcohol Use Disorders Identification Test-Consumption (AUDIT-C) scores (≥3 for women and ≥4 for men), a seven-day drinking diary, and feedback on a readiness to change form to identify people with risky drinking and to inform appropriate advice and feedback. One in four community pharmacy customers (n = 246) offered the alcohol BI were initially interested, and half of these (n = 134, 87 of whom were men) received the intervention. Of the 128 customers whose alcohol use was recorded, 16% (n = 21) were classified as high-risk drinkers, 56% (n = 72) as increasing-risk drinkers, and 27% (n = 35) as low-risk drinkers. Three months following BI, low- and increasing-risk drinkers were contacted by a member of the study team to obtain a post-BI AUDIT-C score and to assess past seven-day alcohol consumption. High-risk drinkers were contacted to ascertain whether they had accessed specialty alcohol services. Seventy-five customers were available for follow-up (response rate, 56%). Of the high-risk drinkers, 91% (n = 10) had seen their general practitioner (GP) and/or accessed specialty alcohol services. Increasing-risk drinkers were found to have significantly reduced their weekly consumption (average decrease, 84%; p = 0.004) and number of drinking days (p = 0.05), however, no significant change in AUDIT-C score was observed. As anticipated, no significant differences in consumption were observed for low-risk drinkers. In this study, community-pharmacy–based alcohol SBI was effective in reducing weekly alcohol use among increasing-risk drinkers and facilitated contact between high-risk drinkers and their GPs and/or specialty alcohol treatment services.

